# Deficiency of Endothelial Nitric Oxide Synthase (eNOS) Exacerbates Brain Damage and Cognitive Deficit in A Mouse Model of Vascular Dementia

**DOI:** 10.14336/AD.2020.0523

**Published:** 2021-06-01

**Authors:** Lulu An, Yi Shen, Michael Chopp, Alex Zacharek, Poornima Venkat, Zhili Chen, Wei Li, Yu Qian, Julie Landschoot-Ward, Jieli Chen

**Affiliations:** ^1^Department of Neurology, Henry Ford Hospital, Detroit, MI-48202, USA; ^2^Department of Neurology, Tianjin Medical University General Hospital, Tianjin, China (Current address).; ^3^Department of Physics, Oakland University, Rochester, MI-48309, USA

**Keywords:** Vascular dementia, endothelial nitric oxide synthase (eNOS), white matter injury, bilateral common carotid artery stenosis (BCAS)

## Abstract

Vascular Dementia (VaD) accounts for nearly 20% of all cases of dementia. eNOS plays an important role in neurovascular remodeling, anti-inflammation, and cognitive functional recovery after stroke. In this study, we investigated whether eNOS regulates brain damage, cognitive function in mouse model of bilateral common carotid artery stenosis (BCAS) induced VaD. Late-adult (6-8 months) C57BL/6J and eNOS knockout (eNOS-/-) mice were subjected to BCAS (n=12/group) or sham group (n=8/group). BCAS was performed by applying microcoils to both common carotid arteries. Cerebral blood flow (CBF) and blood pressure were measured. A battery of cognitive functional tests was performed, and mice were sacrificed 30 days after BCAS. Compared to corresponding sham mice, BCAS in wild-type (WT) and eNOS-/- mice significantly: 1) induces short term, long term memory loss, spatial learning and memory deficits; 2) decreases CBF, increases ischemic cell damage, including apoptosis, white matter (WM) and axonal damage; 3) increases blood brain barrier (BBB) leakage, decreases aquaporin-4 (AQP4) expression and vessel density; 4) increases microglial, astrocyte activation and oxidative stress in the brain; 5) increases inflammatory factor interleukin-1 receptor-associated kinase-1(IRAK-1) and amyloid beta (Aβ) expression in brain; 6) increases IL-6 and IRAK4 expression in brain. eNOS-/-sham mice exhibit increased blood pressure, decreased iNOS and nNOS in brain compared to WT-sham mice. Compared to WT-BCAS mice, eNOS-/-BCAS mice exhibit worse vascular and WM/axonal damage, increased BBB leakage and inflammatory response, increased cognitive deficit, decreased iNOS, nNOS in brain. eNOS deficit exacerbates BCAS induced brain damage and cognitive deficit.

Vascular Dementia (VaD) accounts for nearly 20% of the cases of dementia and is the second leading form of dementia after Alzheimer’s disease (AD). [1] VaD is likely caused by multiple pathological processes, including neurovascular dysfunction and chronic cerebral hypoperfusion [2]. Bilateral common carotid artery stenosis (BCAS) in mice induces chronic cerebral hypoperfusion, white matter (WM) damage, astrogliosis, hippocampal neuronal loss as well as neuro-inflammation and blood brain barrier (BBB) disruption, which in concert, result in cognitive dysfunction[3-5]. Thus, BCAS in the mouse induces parallel underlying pathophysiological alterations evoking dementia as found in patients with VaD [6-9].

Systemic hypertension, a vascular risk factor and a major cause of morbidity and mortality worldwide, is associated with a decline in executive function, speed of cognitive processing, and memory. Hypertension promotes cerebrovascular dysregulation, inflammation, decreased BBB integrity, impaired toxic/metabolic waste clearance (glymphatic function), and small vessel disease [6]. Nitric oxide (NO) produced by endothelial nitric oxide synthase (eNOS) has a crucial role in the regulation of systemic blood pressure, vascular tone, vascular remodeling, and angiogenesis [7, 8]. Aging and cerebrovascular disease are associated with decreased eNOS and increased incidence of cardiac dysfunction and cognitive deficits [9, 10]. Enhanced eNOS phosphorylation also regulates neurogenesis [11] and influences synaptic plasticity [12] and inflammatory response [13]. Genetic inactivation of eNOS causes activation of microglia and promotes a pro-inflammatory phenotype in the brain [14]. Stroke in eNOS knockout (eNOS-/-) mice induces increased cerebral infarction volume and severe neurological and cognitive functional deficit, and reduced long-term potentiation compared to wild-type (WT) mice [15-17]. Aged heterozygous eNOS(+/-) mice exhibit spontaneous thrombotic cerebral infarctions and leading to progressive cognitive impairment[18]. eNOS plays an important role in modulating amyloid precursor protein (APP) expression and microglial activation within the brain and cerebrovasculature, and leads to impairment of spatial memory[19, 20]. Middle aged (14-15 months old) eNOS-/- mice have increased APP, amyloid beta levels, microglial activation as well as impaired spatial memory [20]. However, whether eNOS impacts brain damage in a model of vascular dementia has not been investigated.

In this study, we investigate whether eNOS deficit induces worse brain damage and cognitive deficit in BCAS mice.

## MATERIALS AND METHODS

All experiments were conducted in accordance with the standards and procedures of the American Council on Animal Care and Institutional Animal Care and Use Committee of Henry Ford Health System. Investigators were blinded to the experimental groups to perform cognitive function tests and immunohistochemistry analysis.

### BCAS model and experimental groups

Male, 6-8 months old, C57BL/6 wild type (WT) mice (weight 28-32g,n=12) and eNOS knock-out (eNOS-/-) mice (age 6-8 months, weight 28-32g,n=12) were subjected to BCAS, as previously described [21]. In brief, mice were anesthetized with 4% isoflurane in a chamber for pre-anesthetic, and spontaneously respired with 2% isoflurane in 2:1 N2O:O2 mixture using a facemask connected and regulated with a modified FLUOTEC 3 Vaporizer (Fraser Harlake). Through a midline cervical incision, both the left and right common carotid arteries were exposed and freed from their sheaths. Then, the artery was placed between the loops of the microcoil (the microcoils made of piano wire with an inner diameter of 0.16 mm, pitch 0.50 mm, and total length 2.5mm) just below the carotid bifurcation. The microcoil was twined by rotating it around the artery. After 60 minutes, another microcoil of the same size was entwined around the left common carotid artery [21]. In a previous study, mortality with 0.16mm microcoils was reported as high as 75% and 15% with 0.18mm microcoils at 14 days after BCAS [21]. However, in our experience, BCAS with 0.16mm microcoils induced ~30% mortality at 14 days after BCAS. CBF values recover to ~70% of baseline value in mice with 0.16mm microcoils and remain significantly lower compared to control mice, whereas, CBF recovers to ~80% and no significant difference is detected compared to control group with 0.18mm microcoils at 30 days after BCAS [22, 23]. Thus, using 0.16mm microcoils induces more severe and chronic cerebral hypoperfusion. Reduced CBF is associated with more severe WM injury indicated by an animal model employing 0.16mm microcoil on left CCA and 0.18mm microcoil on right CCA resulting in greater WM injury in the left hemisphere [3]. In addition, after 1 month of hypoperfusion, mice with 0.18mm microcoils do not exhibit infarctions or hemorrhage in gray matter regions, while mice with 0.16mm microcoils exhibit microinfarcts in the hippocampus and in WM which is consistent with our findings [21, 22]. Severe hemodynamic derangement seems to be a direct cause of mortality [24]. Thus, the BCAS model with 0.16mm microcoils induces severe impairment compared to the BCAS model with 0.18mm microcoils at 1 month. Sham mice were subjected to similar procedures but without placing the microcoils. A battery of cognitive tests was employed to evaluate cognition and memory 21-28 days after BCAS. Mice were sacrificed at 30 days after BCAS for immunohistochemical analysis.

### CBF measurement

Regional cerebral blood flow (rCBF) was measured before BCAS, and on days 1, 3, 7, 14 after BCAS using Laser Doppler flowmetry (LDF Peri Flux PF4, Perimed AB) [25]. Briefly, mice were anesthetized as described above, and body temperature was maintained at 37±1.0°C during the measurement period. Using a midline scalp incision, the skull was exposed and non-contact, regional CBF in the both brain hemispheres were continuously recorded for 30 seconds. The data are presented as percentage of baseline values.

### BP measurement

To test whether BCAS and eNOS-/- affects BP, diastolic arterial pressure (DAP), mean arterial pressure (MAP) and systolic arterial pressure (SAP) were measured at 29 days after BCAS using a tail-cuff method (CODA 8-Channel high throughput non-invasive BP system, KENT scientific). The mice were habituated for 5 min in plastic restrainers for 3 consecutive days before experiments were performed. Body temperature was maintained at 37^?^C using a warming pad. Blood pressure was recorded and averaged over 15 consecutive readings.

### Cognitive function tests

Three cognitive function tests including Morris Water Maze (MWM) test, Odor Test and Novel Object Recognition (NOR) test were performed following previously described methods on 21-28 days after BCAS by an investigator blinded to the experimental groups[26-29]. The MWM test was used to evaluate spatial and visual learning and memory to assess hippocampal memory deficits [31]. The Odor Test that evaluates olfactory learning and memory based on animal’s preference for new smells was conducted with a retention delay of 24 hours and used to test long term memory [30]. The NOR test is a relatively low-stress, efficient test to investigate different aspects of learning and memory based on discrimination ability[30].

### Histological and immunohistochemical assessment

Mice were euthanized at 30 days after BCAS (n=8/group). Under deep anesthesia, mouse brain was harvested and fixed with 4% paraformaldehyde for 48 h and then embedded in paraffin wax. A series of coronal sections (6 µm thick) were cut and processed. Hematoxylin and Eosin (H&E) staining was used for lesion volume calculation.

Immunostaining was used to test whether BCAS regulates BBB, vascular and white matter integrity, apoptosis and neuroinflammation. Bielschowsky silver (BS, an axon marker) and luxol fast blue (LFB, a myelin marker) staining were used for quantification of axon and myelin density, respectively. Antibodies against glial fibrillary acidic protein (GFAP, astrocyte marker, Dako, Z0334,1:10,000), Ionized calcium binding adaptor molecule 1 (IBA1, microglia and macrophage marker, Wako,019-19741,1:1000), Albumin (a BBB leakage marker, Abcam,ab53435, 1:500), AQP-4 (Aquaporin-4, EMD Millipore, ab3594, MA, 1:1500), NADPH oxidase 2 (Nox2, BD Biosciences, 611415, 1:400), von Willebrand factor (vWF, Dako, Santa Clara,A0082, 1:400), interleukin-1 receptor-associated kinase-1 (IRAK-1,Santa Cruz Bio, sc-5288, 1:50) and amyloid beta (Aβ, Abcam, ab10148, 1:100) were used. For detecting the apoptotic cells in brain tissue, the extent of cell death was assessed and quantified by TdT-mediated Biotin-dUTP Nick End labeling (TUNEL) stain using a TUNEL kit (Millipore, Billerica, USA).

An investigator blinded to the experimental groups performed quantification analysis. For immunostaining measurements, 8 fields of view of striatum, corpus callosum or hippocampus from each brain, were digitized under a 20× or 40× objective (OlympusBX40) using a 3-CCD color video camera (Sony DXC-970MD) interfaced with an MCID image analysis system (Imaging Research). Data were averaged to obtain a single value for each animal and presented as percentage of positive area or number of positive cells/mm^2^.

### Polymerase Chain Reaction (PCR)

To test cytokine expression, total RNA was isolated with TRIzol (Invitrogen) and 2 µg total RNA was used to make cDNA using M-MLV (Invitrogen), following standard protocol. Then, 2 µl of cDNA was utilized to perform quantitative PCR using the SYBR Green real time PCR method on a ViiA 7 PCR instrument (Applied Biosystems) using 3-stage program parameters provided by the manufacturer, as follows; 2 min at 50°C, 10 min at 95°C, and then 40 cycles of 15 sec at 95°C and 1 min at 60°C. The samples were tested by an investigator blinded to experimental groups. Relative gene expression was analyzed by the 2 -ΔΔCt method. The following primers were used:

GAPDH: FWD: GCCAAGGCTGTGGGCAAGGT; REV: TCTCCAGGCGGCACGTCAGA. Inducible Nitric Oxide Synthase (iNOS): FWD: GTCCTAAGGAAG CTTGTGGATG; REV: GAAATCTTGTGGGACAGA CTCC; Neuronal Nitric Oxide Synthase (nNOS): FWD: GCCACACTTCTCCTCACACA; REV: GGTCCTTCTC CATCTCGGGT; IL-6 FWD: TGATGCACTTGCAG AAAACA; REV: ACCAGAGGAAATTTTCAATAGGC; IRAK4: FWD: GTTGGCGACCTTGTGGATCT; REV: TAGGAGGCAGGCTTTTGACG

### Statistical analysis methods

Unpaired 2-tailed Student t test was used for testing significance of 2 groups with Graph Pad Prism 8 (Graph Pad Software Inc., San Diego, CA). One-way Analysis of Variance (ANOVA) was utilized for functional outcome. *P < 0.05 was considered statistically significant. Data in all figures are presented as mean ± SEM.

**Figure 1.**
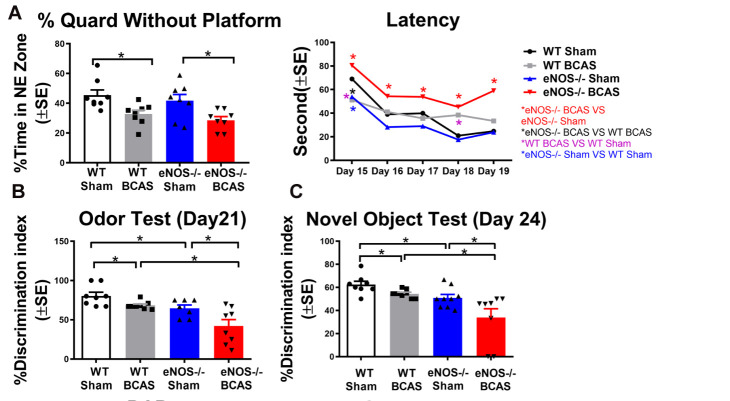
BCAS significantly induces cognitive dysfunction compared to WT-sham mice. eNOS-/-BCAS mice exhibit severe cognitive function impairment compared to eNOS-/-sham or WT-BCAS mice, respectively. (A) Water Maze test; Latency: *p<0.05. (B) Odor Test; (C) Novel Object Test.

## RESULT

### Compared to WT-sham, eNOS-/-sham and WT-BCAS significantly induces memory deficits; eNOS-/-BCAS mice exhibit exacerbated cognitive impairment compared to eNOS-/-sham and WT-BCAS mice

In this study,14-day mortality rate was 33% (4/12) in WT-BCAS and in eNOS-/-BCAS mice (Fig. 1). There was no mortality in WT-sham and eNOS-/-sham groups. To test whether the BCAS impairs cognitive function in mice, short-term memory, long-term memory, spatial learning and memory were evaluated 21-28 days after BCAS. Figure 1A shows that BCAS in WT mice significantly impairs spatial learning and memory as indicated by significantly increased escape latency to hidden platform as well as reduced percentage of time spent in the target platform quadrant in the MWM test. BCAS in WT-sham and eNOS-/-sham mice also significantly decreases discrimination index in both NOR test and Odor Test, indicating poor short- and long-term memory deficits compared to WT-sham mice, as shown in Figure 1B-C. eNOS-/-BCAS mice exhibit significantly worse cognitive dysfunction compared to eNOS-/-sham or WT-BCAS mice, respectively. The data indicate that BCAS significantly induces short-term, long-term, spatial learning and memory deficits in mice and eNOS deficiency exacerbates BCAS induced cognitive impairment.

### eNOS deficit significantly increases blood pressure compared to WT-sham or WT-BCAS mice, respectively; BCAS significantly decreases CBF, and eNOS deficiency exacerbates CBF decline after BCAS

**Figure 2.**
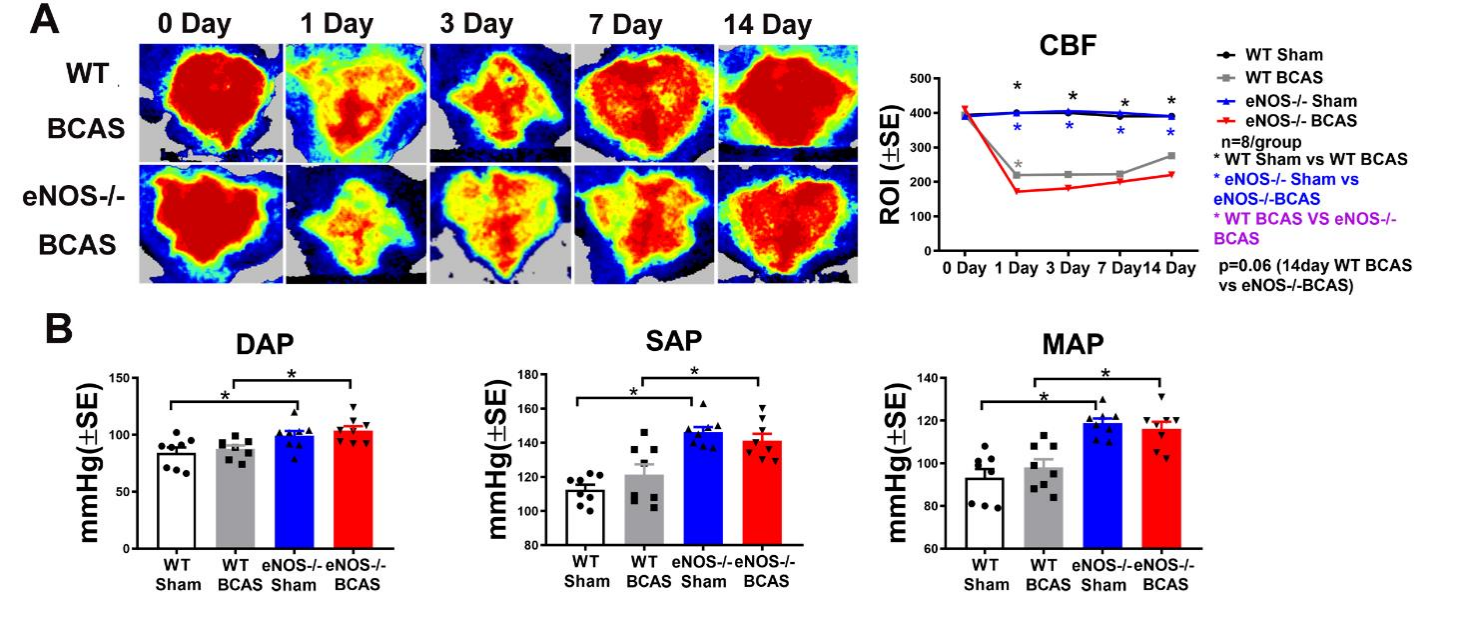
BCAS significantly decreases CBF compared to WT-sham or eNOS-/-sham control mice, respectively. eNOS-/-BCAS mice exhibit significantly decreased CBF at 1 and 14 days after BCAS compared to WT-BCAS mice. eNOS-/-sham and eNOS-/-BCAS significantly increases SAP, DAP and MAP compared to WT-sham or WT-BCAS mice, respectively. (A) CBF measurement and qualification; (B) DAP, SAP and MAP measurement and quantification data. *p<0.05, n=8/group.

To test whether BCAS or eNOS deficit regulates BP and CBF, BP was measured using the tail-cuff method. CBF was measured on days 0, 1, 3,7and 14 after BCAS (n=8/group) (Fig. 2). Figure 2A shows that BCAS significantly decreases CBF at 1, 3, 7 and 14 days after BCAS compared to WT-sham group. eNOS-/- BCAS mice exhibit significantly decreased CBF compared to WT-BCAS mice on day 1 (p<0.05) and day 14 (p=0.06). Figure 2B shows that BCAS did not affect BP in WT mice, while eNOS-/-sham and eNOS-/-BCAS mice exhibit significantly increased systolic arterial pressure (SAP), diastolic arterial pressure (DAP) and mean arterial pressure (MAP) compared to WT-sham and WT-BCAS mice, respectively. The data indicate that eNOS deficit increases BP compared to WT mice. However, eNOS-/-BCAS did not increase blood pressure compared to eNOS-/-sham control. BCAS decreases CBF, and eNOS deficit exacerbates BCAS-induced hypoperfusion compared to WT-BCAS.

### BCAS significantly increases brain damage, apoptosis, and axonal/WM damage compared to WT-sham mice; eNOS-/-BCAS mice exhibit significantly increased lesion volume, apoptosis and severe axonal/WM damage compared to eNOS-/-sham or WT-BCAS mice, respectively

**Figure 3.**
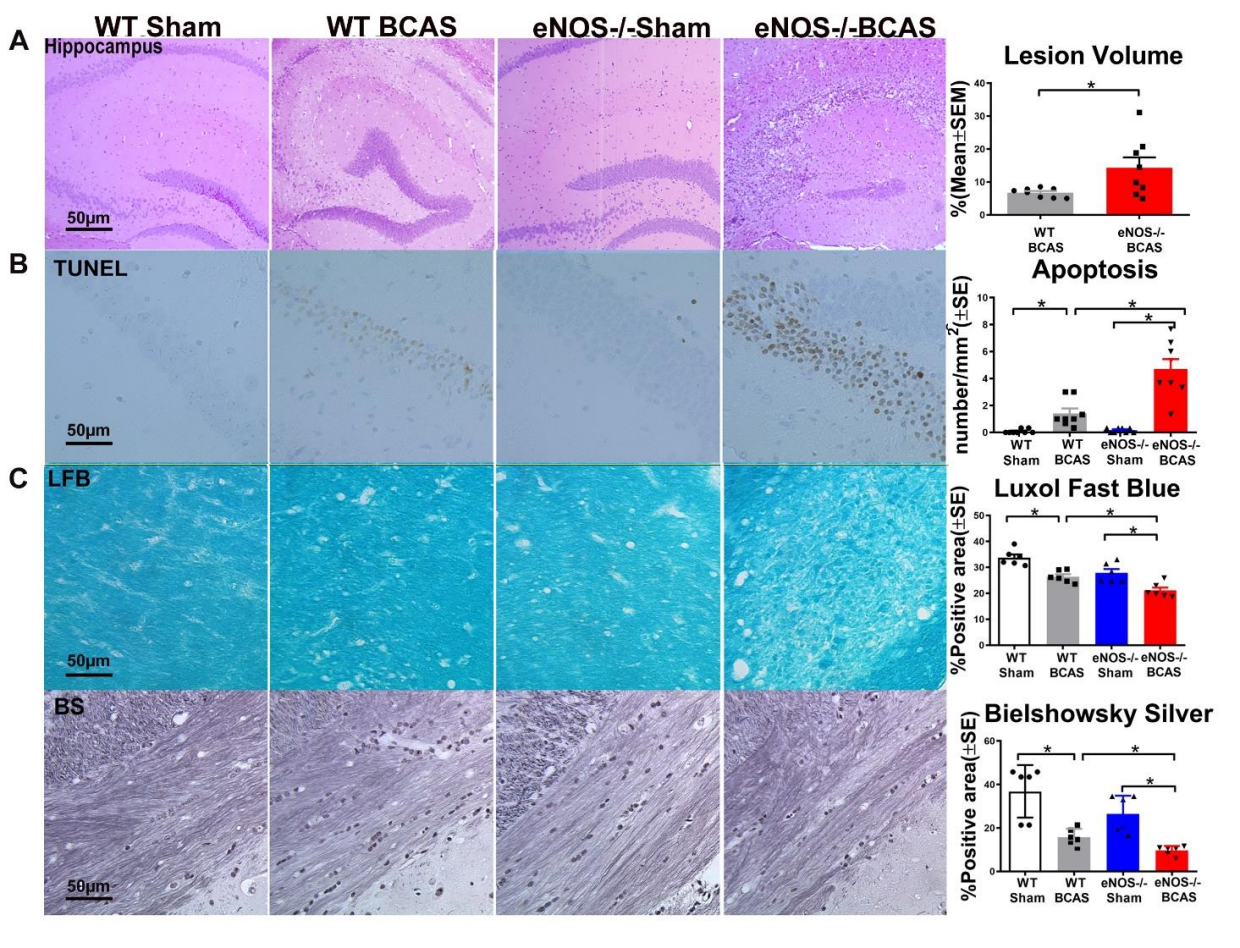
BCAS significantly increases brain lesion volume, apoptosis and WM/axonal damage compared to WT-sham mice. eNOS-/-BCAS mice exhibit significantly increased lesion volume, apoptosis and WM/axonal damage compared to eNOS-/-sham or WT-BCAS mice, respectively. (A) HE staining and quantification data; (B) Tunel staining and quantification data; (C) LFB and BS staining and quantification data. *p<0.05; HE staining: n=8/group; Tunel: n=8/group; LFB and BS: n=6/group.

To test whether BCAS induces cerebral ischemic damage, H&E staining was employed to calculate lesion burden. Figure 3A shows that BCAS in WT mice induces ischemic damage and eNOS-/-BCAS mice exhibit increased lesion volume compared to WT-BCAS mice (Fig. 3).

To evaluate whether BCAS induces brain cell apoptosis, Tunel staining was performed. Figure 3B shows that WT-BCAS significantly increases apoptotic cell numbers in the corpus callosum compared to WT-sham mice. eNOS-/-BCAS mice exhibit increased apoptosis compared to eNOS-/-sham or WT-BCAS mice, respectively. These data indicate that BCAS induces apoptosis in brain and eNOS deficit exacerbates BCAS-induced apoptosis in brain.

To evaluate the effects of BCAS on WM and axonal damage, LFB and BS staining were employed. Figure 3C shows that BCAS significantly decreases myelin (LFB staining) and axon (BS staining) density in the WM tracts of corpus callosum and WM bundles in the striatum compared to WT-sham mice. eNOS-/-BCAS mice exhibit significantly exacerbated WM/ axonal damage compared to eNOS-/-sham or WT-BCAS mice, respectively. The data indicate that BCAS induces axonal/WM damage and eNOS deficit exacerbates BCAS-induced WM/axonal injury.

### WT-BCAS and eNOS-/-sham mice exhibit significantly increased disruption of the BBB and vascular damage compared to WT-sham mice; eNOS-/-BCAS mice exhibit significantly increased BBB disruption and vascular damage compared to eNOS-/-sham or WT-BCAS mice, respectively

**Figure 4.**
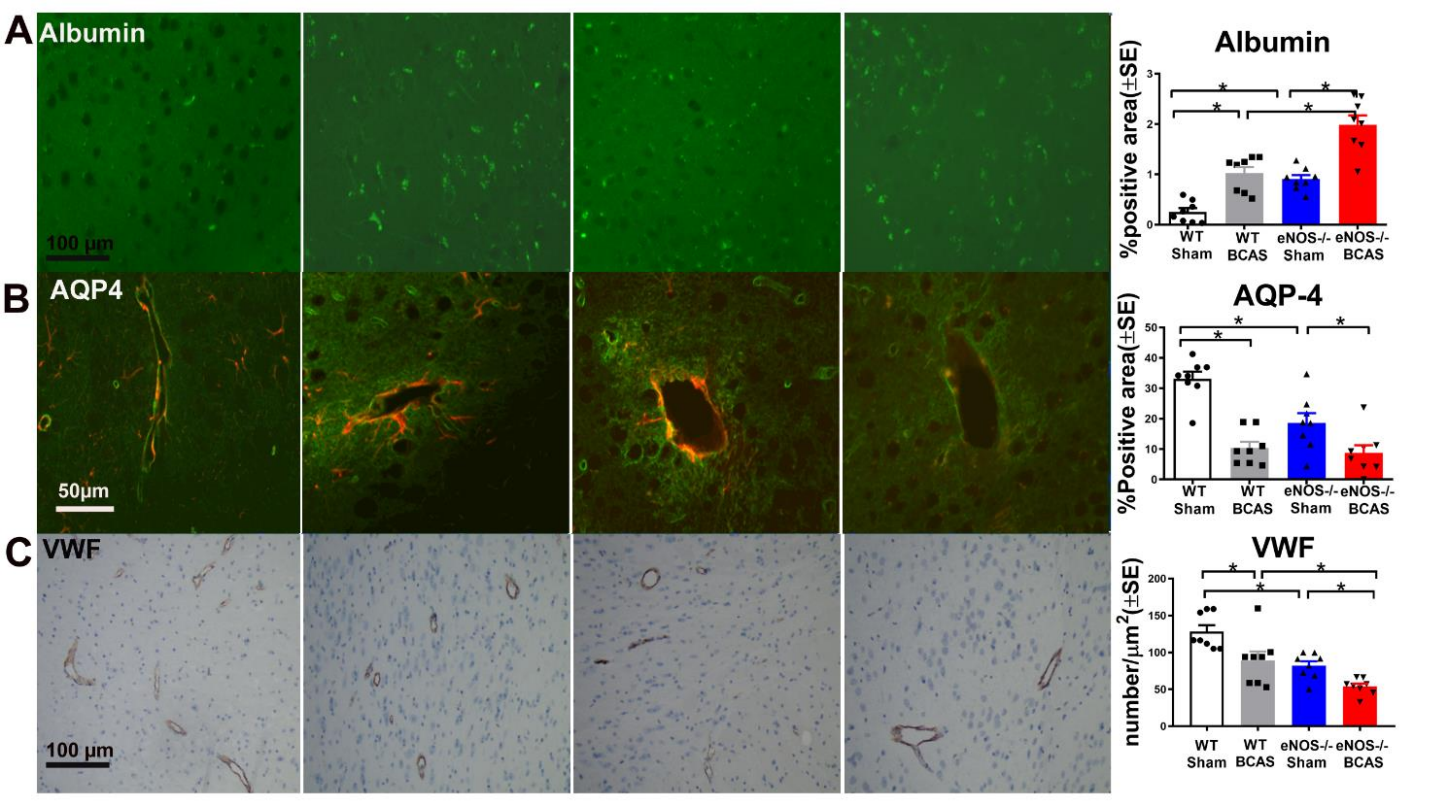
BCAS significantly induces BBB leakage and decreases AQP4 expression and vessel density compared to WT-sham mice. eNOS-/-BCAS exhibits severe BBB leakage and decreased vessel density compared to eNOS-/-sham or WT-BCAS mice, respectively. eNOS-/-BCAS significantly decreased AQP4 expression compared to eNOS-/-sham control mice. (A) Albumin; (B) AQP-4; (C) vWF staining and quantification data; *p<0.05, n=8/group.

To test if BCAS induces BBB disruption, immune-fluorescence staining of albumin was performed (Fig. 4). Figure 4A shows that BCAS significantly increases BBB leakage identified by increased albumin expression in brain, while eNOS deficit significantly increases BBB leakage compared to WT-BCAS mice. To investigate whether BCAS induces vascular damage, vWF and AQP-4 expression were evaluated. Figure 4B-C shows that AQP-4 expression around blood vessels and vessel density significantly decreased in WT-BCAS mice compared to the WT-sham group, while eNOS-/-BCAS mice exhibit significantly decreased vessel density and AQP4 expression. The data indicate that BCAS induces BBB leakage, decreases vessel density and AQP4 expression, while eNOS deficit exacerbates BCAS-induced vascular damage.

### BCAS increases inflammation and oxidative stress in brain compared to WT-sham mice; eNOS-/-BCAS mice exhibit significantly enhanced inflammation and oxidative stress response compared to eNOS-/-sham or WT-BCAS mice, respectively

**Figure 5.**
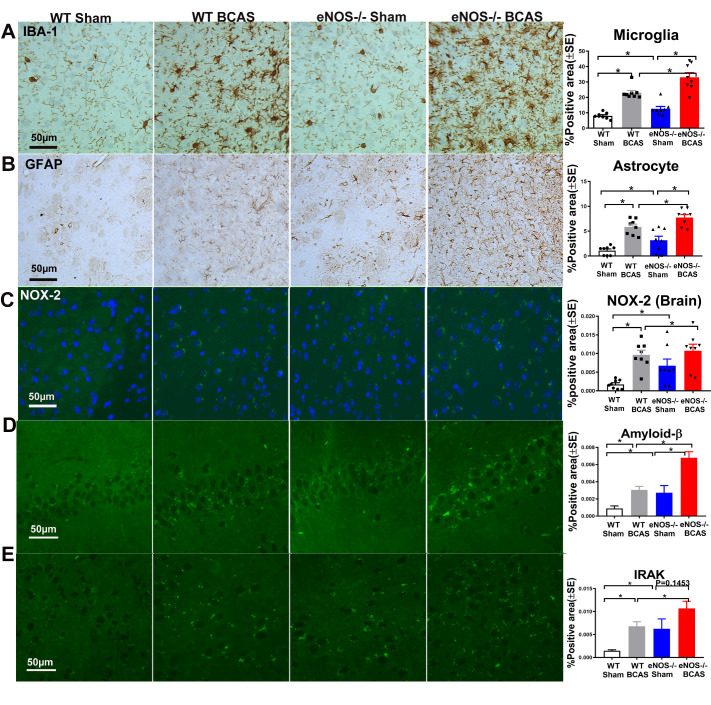
BCAS significantly increases microglial and astrocyte expression, NOX-2, IRAK1 and Aβ expression in brain compared to WT-sham mice. eNOS deficit significantly increases microglial and astrocyte activity, NOX-2, IRAK1 and Aβ expression in brain compared to WT-sham control mice; eNOS-/-BCAS exhibits increased expression of microglial, astrocyte, NOX-2, Aβ and IRAK1 expression compared to eNOS-/-sham or WT-BCAS mice, respectively. (A) IBA-1; (B) GFAP; (C) NOX-2; (D) Amyloid-β; (E) IRAK1. *p<0.05, n=8/group.

To test whether BCAS triggers astrocyte and microglial activation in the brain, GFAP and IBA1 immuno-histochemistry, respectively, was performed (Fig. 5). Figure 5A-B shows that BCAS significantly increases the expression of GFAP (marker for astrocyte) and IBA1 (marker for microglia) compared to WT-sham mice. eNOS-/-BCAS mice exhibit significantly increased expression of GFAP and IBA1 compared to eNOS-/-sham mice or WT-BCAS mice, respectively.

NOX-2 immunostaining was employed to investigate oxidative stress response after BCAS. Figure 5C shows BCAS significantly increases NOX-2 expression in brain compared to WT-sham mice. eNOS-/-BCAS increases NOX-2 expression compared to WT-BCAS mice. No differences in NOX-2 were detected between eNOS-/- BCAS and eNOS-/- sham group (p=0.22). The data indicate that BCAS triggers inflammation and oxidative stress response in brain, and eNOS deficit exacerbates BCAS-induced inflammation and oxidative stress response.

By immunostaining, we found that BCAS significantly increases Aβ and IRAK1 expression in brain compared to WT-sham mice. eNOS-/-BCAS increases Aβ and IRAK1 expression compared to WT-BCAS mice. There’s no significance in IRAK1 between eNOS-/-sham and eNOS-/-BCAS mice showed by Figure 5D-E.

### BCAS significantly decreases iNOS and nNOS expression in brain and increases IRAK4, IL-6 compared to WT-sham mice; eNOS-/-BCAS mice exhibit significantly decreased iNOS, nNOS and increased IL-6, IRAK4 expression compared to WT-sham or WT-BCAS mice, respectively

**Figure 6.**
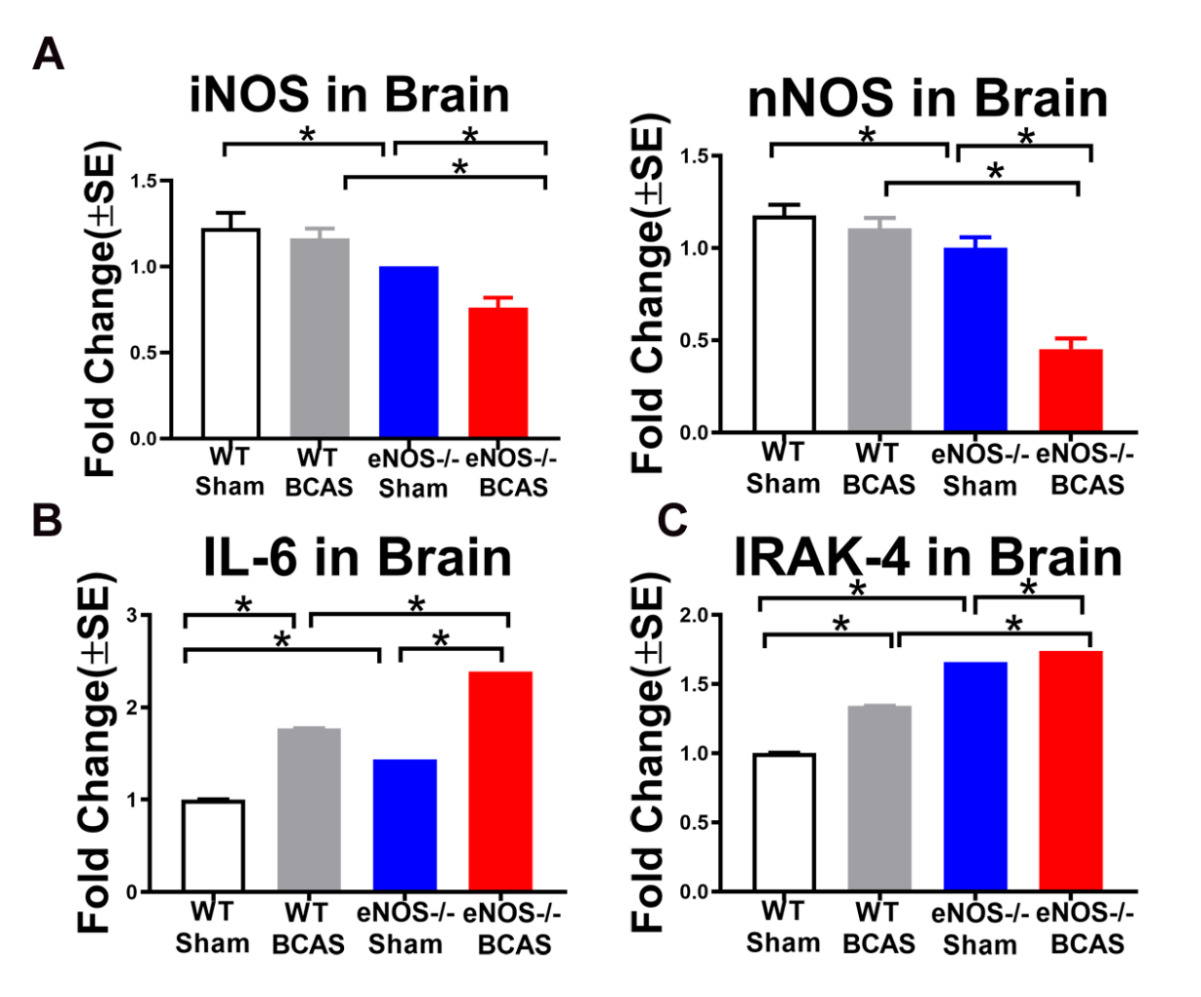
BCAS significantly increases IL-6 and IRAK4 compared to WT-sham mice. eNOS-/-BCAS mice shows decreased iNOS and nNOS expression compared to WT-BCAS or eNOS-/-sham mice. (A) iNOS and nNOS; (B) IL-6; (C) IRAK4 gene expression. n=6/group; *p<0.05.

To test the effect of BCAS and eNOS deficiency on iNOS and nNOS levels in brain, PCR was employed (Fig. 6). Data in Figure 6A indicate that BCAS in WT mice does not significantly alter iNOS and nNOS levels. Compared to WT sham mice, sham eNOS-/- exhibit significantly decreased iNOS and nNOS expression. eNOS-/-BCAS mice exhibit significantly decreased iNOS or nNOS levels compared to sham eNOS-/- mice as well as WT-BCAS mice. Figure 6B-C shows that BCAS significantly increases IL-6 and IRAK4 expression in brain compared to WT-sham mice while eNOS-/-BCAS significantly increases IL-6 and IRAK4 expression compared to WT-BCAS mice or eNOS-/-sham mice, respectively. Our data indicate that eNOS deficiency suppresses iNOS and nNOS in brain. BCAS increases IL-6 and IRAK4 expression in brain, while eNOS deficiency exacerbates these results.

## DISCUSSION

In this study, we found that BCAS significantly decreases CBF and vessel density, and induces cognitive deficits, ischemic lesions, apoptosis, BBB leakage, WM/axonal damage, and inflammation in brain. These findings are consistent with previous BCAS studies [31]. We also found that eNOS deficiency significantly increases BP and aggravates BCAS induced hypoperfusion, cognitive deficits and pathological changes in the brain which may be mediated by aggravating inflammatory responses.

### eNOS deficit increases BP and exacerbates BCAS induced hypoperfusion and cognitive dysfunction

eNOS is the primary isoform of NOS and plays an important role in NO regulation of physiological functions[32]. eNOS/NO is a key paracrine regulator of vascular tone, vascular smooth-muscle proliferation and inhibits platelet aggregation and leukocyte adhesion, as well as regulates blood pressure [33, 34]. eNOS deficient mice are hypertensive and exhibit significantly increased lesion volume and reduced CBF after stroke compared to WT-stroke mice [15]. eNOS deficit decreases vasodilation and increases BP, as well as impairs vascular remodeling following hind limb ischemia [35]. A clinical long-term follow-up study for a median 6.9 years concluded that cerebral hypoperfusion is associated with accelerated cognitive decline and an increased risk of dementia in the general population [36]. Consistent with previous findings [31, 37], our data indicate that BCAS induces long-term cerebral hypoperfusion, ischemic lesions, and impairs short-term, long-term and spatial learning and memory. Deficiency of eNOS increases BP and exacerbates BCAS induced hypoperfusion, ischemic lesion volume and cognitive dysfunction compared to WT-BCAS mice. At baseline, there were no significant differences in CBF between WT and eNOS-/- mice. Consistent with our findings, it has been previously reported that under normal conditions, resting regional CBF was not significantly different between WT, nNOS deficient, and eNOS deficient mice [38-40]. Resting CBF values are maintained within the physiologic range after eNOS gene deletion most likely due to compensatory mechanisms of CBF regulation and compensatory action of perivascular neuronal NOS [38-40].

We also fsound that in response to BCAS, the CBF reduction in eNOS-/- mice is greater than in WT mice. Similarly, after traumatic brain injury, CBF reduction was significantly greater in eNOS-/- mice compared to WT mice at 24 hours after injury [41]. In mice subjected to BCAS and treated with bone marrow mononuclear cells (BMMNC) at 24 hours after BCAS, early CBF recovery and protection against WM injury were associated with an increase in Ser1177 phosphorylated-eNOS levels[42]. Administering an NOS inhibitor (L-NAME) significantly attenuated BMMNC induced CBF recovery in BCAS mice as well as decreased CBF in vehicle treated BCAS mice, indicating that eNOS dependent pathways are involved in rapid autoregulatory mechanisms after ischemic injury to the brain [42]. Our data show that eNOS-/- BCAS mice exhibit poor CBF recovery at 7 days after BCAS, while eNOS-/- sham mice exhibit no difference compared to WT sham mice. It is likely that eNOS dependent pathways play a role in early CBF recovery.

### eNOS deficiency exacerbates BCAS induced BBB leakage and vascular damage

Progressive BBB dysfunction becomes a feedback loop that gives rise to cognitive impairment and onset of dementia [26, 43]. BBB breakdown also contributes to reduced vessel density and hypoperfusion [44]. In addition, astroglial water channel aquaporin-4 (AQP-4) regulates the glymphatic system [45], a brain-wide network of perivascular pathways that facilitates the exchange of interstitial and cerebrospinal fluid to support the clearance neurotoxin from the brain interstitium which can otherwise contribute to dementia [26, 46, 47]. In our study, we found that mice subjected to BCAS exhibit significantly increased BBB leakage, decreased vessel density, decreased AQP4 expression around cerebral vessels, which, were exacerbated in eNOS-/- BCAS mice. eNOS regulates BBB leakage and vascular function and eNOS/NO is a key regulator of vascular homeostasis [48]. NO production by vascular endothelium maintains vessel integrity, angiogenesis and maintenance of CBF in ischemic-reperfusion model or brain injury [15, 41, 49]. Inhibition of NO production by vascular endothelium leads to increased microvascular permeability and reduces CBF after brain injury or stroke [15, 41]. In our study, we found that eNOS deficit significantly increases BCAS-induced BBB leakage and vascular dysfunction damage, which may contribute to exacerbated cognitive dysfunction observed in eNOS-/-BCAS mice.

### eNOS deficiency exacerbates BCAS induced axonal/WM damage in mice

Hypoperfusion leads to cellular apoptosis, ischemic cell damage, and WM rarefaction and diffuse damage in WM tracts [21]. Slowed or desynchronized impulse conduction between distant cortical regions resulting from defects in myelin insulation leads to impaired cognitive ability. Extensive WM damage such as vacuolization, rarefaction, and demyelination in the periventricular region has been reported in VaD patients[50, 51]. Such periventricular WM damage disrupts neuronal connections to the frontal lobe and may be central to VaD induced cognitive impairment[52]. eNOS is known to regulate neurogenesis, axonal outgrowth, and synaptic plasticity in hippocampus and cortex, thereby, participating in basic memory formation [14, 53, 54]. eNOS in the microvasculature generates the tonic levels of NO that influence axonal function [55]. eNOS deficit significantly increases WM/axon damage after stroke compared to WT-stroke mice, and the axon density is significantly positive correlated with neurological functional recovery [54]. In our study, we found that eNOS-/- mice subjected to BCAS exhibit significantly increased WM/axon damage which may also contribute to worse cognitive function compared to WT BCAS mice.

### eNOS deficiency exacerbates BCAS induced inflammation and oxidative stress in mice

Hypoperfusion impacts microglial/astrocyte activity [34-36]. Activated astrocytes release a variety of reactive oxygen species (ROS) and proinflammatory cytokines and form glial scars, causing severe damage to neural cells and adversely affects cognitive function [56]. Microglial activation has been shown to exacerbate neuro-inflammation and WM/axonal damage as well as reduce synaptic plasticity, which contribute to depressive-like behavior and eventual cognitive decline[57]. NOX-2, a subunit of nicotinamide adenine dinucleotide phosphate oxidase, is one of the major sources of oxidative stress and can regulate microglia activation and increase demyelination[58-61]. Oxidative stress induces release of pro-inflammatory cytokines, which in turn causes cellular apoptosis, BBB disruption and creates a hostile environment for neural repair[62]. The eNOS/NO pathway inhibits leukocyte adhesion and promotes pro-inflammatory cytokine gene expression[63]. eNOS null mice exhibit consistent upregulation in inflammatory pathways [64] and increased macrophage infiltration in carotid arteries [65]. Our data indicate that BCAS induces significant glial activation and increases NOX-2 expression in brain which are exacerbated in eNOS-/- BCAS mice.

Endogenous production of NO is catalyzed by NOS. iNOS is reduced in hippocampus of adult spontaneously hypertension dementia rats [66]. Reduction of nNOS in the amygdala showing severe Lewy pathology in patience [68]. When eNOS becomes dysfunctional, nNOS expression also decreases [69]. In our study, there were no differences in iNOS and nNOS levels between WT-sham and WT-BCAS at 1 month after BCAS which suggest that iNOS and nNOS do not participate in WT early stage of VaD. However, a significant reduction in iNOS and nNOS was observed in eNOS-/- sham and eNOS-/-BCAS mice compared to WT-sham and WT-BCAS mice, respectively, indicating that eNOS knockout influences iNOS and nNOS transformation and expression. Several studies have reported that administration of NO donors exerts significant neuroprotective effects, reduces lesion volume and improves CBF following ischemic stroke [70, 71]. Studies have also indicated that inhalation of NO depending on concentration and duration of inhalation, can increase CBF by dilating arterioles in the penumbra to improve collateral blood flow, which in turn improves neurological functional outcome after experimental stroke [72, 73]. Treatment with NO donor agents such as L-Arginine, significantly increases CBF and decreases infarct volume via NO-mediated mechanisms in normotensive and hypertensive rats subjected to stroke [74]. Sub-group analysis revealed that in a large eNOS trial, transdermal glyceryl trinitrate administered within 6 hours of stroke onset significantly improves neurological function, decreases deaths, lowers blood pressure and improves cognitive outcome at 90 days compared to non-treated control group [75]. In aging as well, NO production is decreased and is associated with adverse cardiovascular outcome [76]. Treatment of aged rats with NO donor molsidomine significantly improves cognitive outcome in passive avoidance test and novel object recognition test [77]. However, NO donor may not only mediate eNOS effect, and may also impact the biological effects of iNOS and nNOS [78, 79].

### eNOS-/-BCAS exacerbates increased IRAK expression and Aβ deposition in brain

IRAK1 is a key mediator via TLR/IL-1R (TIR) signaling which initiates diverse downstream pathways and a cascade of events that increase pro-inflammatory transcription factors [80, 81]. Microglia can be activated to polarize to M2 proinflammatory phenotype by increasing IRAK1 expression which induces neuroinflammation and deposition of Aβ[82, 83]. Activated IRAK1 signal by infection promotes accumulation of Aβ and chronic inflammation[84]. Aβ interacts with the cell membrane, alters calcium homeostasis which changes cellular permeability, mitochondrial dysfunction, and inflammation [85]. Knockdown IRAK4 in microglia significantly inhibits the downstream production of inflammatory mediators [86], promotes amyloid clearance in a murine model of AD [87], as well as restores olfactory behavior [87]. IRAK4 regulates IL-6, which is an inflammatory cytokine that mediates innate and adaptive immunity [88]. Circulating inflammatory factors, such as IL-6, are significantly increased before clinical onset of dementia and predict cognitive decline [89]. IL-6 is significantly increased in moderate-severe AD and VaD patients [90]. Aβ impairs eNOS activity and induces vascular dysfunction and cognitive deficit [91]. Aged eNOS heterozygous knockout (eNOS^+/-^) mice exhibit significantly increased cerebrovascular concentration of Aβ40 [92]. eNOS deficit significantly increases protein levels of Aβ protein precursor in brain tissue compared with WT control [19]. Inhibition of eNOS with the specific NOS inhibitor L-NAME (N(G)-nitro-l-arginine methyl ester) triggers human brain microvascular endothelial cells to increase generation of Amyloid precursor protein (APP) as well as increases secretion of the Aβ in vitro [19]. In addition, blood vessel pulsations are believed to stimulate flow of interstitial fluid as an Aβ clearance mechanism [93]. Loss of eNOS significantly contributes to stiffening of cerebral blood vessels thereby exerting a detrimental effect on clearance of Aβ [14, 94]. Our data show that BCAS and eNOS deficit significantly increases IRAK1 expression and Aβ deposition, with maximum IRAK1 and Aβ observed in eNOS-/-BCAS group which may contribute to eNOS-/-BCAS induced worse cognitive impairment.

### Limitation

We are aware that age and gender also affect VaD. Aging is a major risk factor for VaD and VaD is prevalent among the older population[95]. However, using aged mice is challenging due to high mortality rate after BCAS in aged WT and eNOS-/- mice. Sex is a biological variable that is frequently ignored in animal study designs, leading to an incomplete understanding of potential sex-based differences in basic biological function. Diffuse brain WM damage has been clinically associated with VaD in both men and women[96, 97]. Sex may moderate the relationships between depressive symptoms and WM lesions [98]. Future studies to test effects of gender differences in BCAS model and effect of eNOS in modulating VaD are warranted.

### Conclusion

BCAS induces cerebral hypoperfusion, vascular and WM/axonal damage, increases BBB leakage, oxidative stress and inflammatory response as well as cognitive deficit. Deficiency of eNOS significantly increases blood pressure and exacerbates BCAS-induced cerebral damage and inflammation.
